# Optimization of [^11^C]methionine PET study: appropriate scan timing and effect of plasma amino acid concentrations on the SUV

**DOI:** 10.1186/2191-219X-3-27

**Published:** 2013-04-15

**Authors:** Kayako Isohashi, Eku Shimosegawa, Hiroki Kato, Yasukazu Kanai, Sadahiro Naka, Koichi Fujino, Hiroshi Watabe, Jun Hatazawa

**Affiliations:** 1 Department of Nuclear Medicine and Tracer Kinetics, Osaka University Graduate School of Medicine, 2-2, Yamadaoka, Suita City, Osaka 565-0871, Japan

**Keywords:** Methionine, PET, Normal volunteers, Plasma concentrations of neutral amino acids

## Abstract

**Background:**

[^11^C]methionine (MET) has been used to monitor amino acid metabolism in tumors, the pancreas, liver, and myocardium. The aim of the present study was to standardize [^11^C]MET positron emission tomography (PET) by optimizing the timing of initiation of the scan and applying correction to the plasma concentrations of neutral amino acids (NAAs), where necessary.

**Methods:**

Sequential whole-body MET PET/computed tomography (CT) was performed in 11 normal adults after they had fasted for at least 4 h. After whole-body CT for attenuation correction and intravenous bolus injection of MET, the subjects were scanned from the parietal to the groin. The scanning was repeated six to seven times. Decay of radioactivity during the PET scan was corrected to the time of initiation of the first scan. The standardized uptake values (SUVs) were evaluated in various organs by setting regions of interest on the tomographic images. Plasma concentrations of NAAs were examined in relation to the SUV values.

**Results:**

The SUVs in the pancreas reached their plateau from 6.5 to 11 min after the MET injection, and in the brain, lung, and myocardium, they reached their plateau from 19.6 to 24.1 min. The MET uptake in the spleen and kidney peaked early after the injection and steadily decreased thereafter. The SUVs in the liver and stomach wall rapidly increased during the first 0 to 4.5 min and gradually elevated thereafter during the scan period. Urinary radioactivity in the bladder reached its plateau from 26.1 to 30.6 min after the MET injection. There were no correlations between the plasma concentrations of NAAs and the maximal SUV in any organs.

**Conclusions:**

The present study revealed the times taken to reach the plateau of MET uptake in various important organs, and little effects of the plasma neutral amino acid concentrations on the SUVs in PET studies conducted after the patients had fasted for at least 4 h. In the MET PET study, 4 h fasting period before MET administration and the scan initiation 20 min after MET administration provide the SUV values independent of scan initiation time and the plasma neutral amino acid concentrations.

## Background

l-Methyl-[^11^C]methionine (MET) is a useful radiotracer in positron emission tomography (PET) conducted for the diagnosis of tumors [1-20]. In particular, MET PET has been shown to enable reliable diagnosis of brain tumors because of the low physiologic uptake of MET in the brain. It has been reported that the extent of tumor cell invasion can be detected more clearly by MET PET than by computed tomography (CT) or MRI [2-5,21]. MET PET has also been used to evaluate amino acid metabolism in the pancreas and liver and, recently, also in the myocardium [22-26]. However, the study protocol for MET PET has not yet been standardized. For example, the reported scan initiation time after MET injection is variable, ranging from 15 to 30 min (Table 1) [1-13,18,24,25]. Moreover, the concentrations of neutral amino acids (NAAs) in the plasma were not measured, despite the possibility of these affecting the tracer MET uptake by tissues in a competitive way. Although the NAA concentrations in the plasma were found to be influenced when measured after a meal, a fasting period before MET PET study has not been suggested. The purpose of this study was to confirm previous published protocols and standardize the scan initiation times of MET PET for various organs. We also examined the effects of the NAA concentrations in the plasma on the standardized uptake values (SUVs) in the MET PET study.

**Table 1:** Summary of the scan initiation times after intravenous injection of MET

**Study**	**Site**	**Fasting before the scan (h)**	**Scan initiation time (min)**	**Dose of MET (MBq)**
Nariai et al. [2]	Brain	No	20	250 to 500
Shinozaki et al. [3]	Brain	≧4	20	370 to 720
Mahasittiwat et al. [4]	Brain	No	23	740
Coope et al. [5]	Brain	No	20	740
Hasebe et al. [6]	Head and neck	≧4	23	374 to 870
Jang et al. [7]	Thyroid	≧6	30	740
Herrmann et al. [8]	Parathyroid gland	3	15 to 20	272 to 603
Morooka et al. [23]	Cardiac tissue	6	20	370
Kanegae et al. [9]	Lung	≧5	15 to 20	361 to 607
Hsieh et al. [10]	Lung	≧6	15 to 30	296 to 555
Lindholm et al. [11]	Breast	≧4	20 to 35	220 to 370
Sasaki et al. [12]	Thymus	≧6	15	70 to 818
Syrota et al. [19]	Pancreas	No	6 to 8	370 to 740
Otsuki et al. [22]	Pancreas	6	30	370 to 740
Koizumi et al. [13]	Pelvis	≧6	23	740
Shiiba et al. [1]	Prostate	Time unknown	20	3 MBq/kg
Dankerl et al. [18]	Bone marrow	5 to 8	20	1,000 ± 0.2 (SD)

## Methods

### Subjects

A total of 11 healthy volunteers (Japanese, nine males and two females) participated in the present study after receiving a detailed explanation on the radiotracer drug and the purpose and contents of the study. This study was performed with the approval of the institutional ethics committee for clinical research of Osaka University. Written informed consent was obtained from all the subjects.

The mean age of the 11 subjects was 24.4 years (range, 24 to 26 years), and the mean height and weight were 168.45 cm (range, 154 to 176 cm) and 59.45 kg (range, 50 to 78 kg), respectively. None of the subjects had a prior history of any major medical illness.

### Administration of the MET and PET imaging protocol

All subjects fasted for at least 4 h before the radiotracer injection and underwent blood laboratory tests for NAA densitometry and sequential whole-body MET PET-CT (Gemini GXL, Philips, Cleveland, OH, USA) in the three-dimensional acquisition mode. The scanner provides 220 continuous transaxial slices with a spatial resolution of 5.3 mm full width at half maximum in the axial direction. The axial field of view is 18 cm. Whole-body low-dose CT for attenuation correction was performed at the beginning of each imaging session. Thereafter, MET (370 MBq/50 kg) was injected as a bolus into the antecubital vein. Repeated whole-body PET scan of the marked area was initiated at the time of the tracer injection. Seven repeated whole-body PET scans from the parietal crown to the groin were performed in each of ten healthy volunteers, and six repeated whole-body PET scans were performed in one healthy volunteer. The data consisted of nine-frame scan with 30-s acquisition time for each frame, and the interval between scans was set at 122 s. Decay of radioactivity during PET scan was corrected to the time of initiation of the first scan. PET reconstruction algorithms were the line-of-response row-action maximum likelihood algorithm (RAMLA) and 3D RAMLA.

### Evaluation of the biodistribution

The radioactivities in various source organs were obtained from reconstructed PET images by averaging the activities (cps/ml) in the regions of interest (ROIs) in each organ since the radioactivity distribution within an organ can be considered uniform [27]. ROIs were manually located on each organ by tracing the MET activity on the PET images, and the maximal standardized uptake value (SUV max) at every mid-scan time was evaluated in various organs by setting ROIs on the whole-body PET-CT images. All the PET counts were corrected for physical decay of ^11^C (T1/2 = 20.4 min). Circular ROIs with a diameter 16 to 32 mm were drawn on tomographic images within the brain, parotid, lung, myocardium, ventricular blood pool, stomach, spleen, liver, pancreas, right kidney, right and left intestine, the urine in the urinary bladder, muscle, and bone marrow, and on tomographic images within the prostate in the male subjects and the breast and corpus uteri in the female subjects. Regarding the bone marrow, the ROI was set and measured to the fifth lumbar body that an anatomical position tends to identify. Time-activity curves in 18 source organs were obtained from the six or seven repeated whole-body PET measurements.

### Measurement of NAA concentrations in the plasma

In all subjects, the NAA concentrations in the plasma were measured after the subjects had fasted for 4 h before the PET study. The venous sampling just before the PET study was submitted and measured by the SRL company. The amino acids measured were methionine, phenylalanine, tryptophan, isoleucine, leucine, valine, threonine, tyrosine, and histidine, which are transported through the same carrier termed the L-system, as methionine [28]. The affinities of the carrier system for these nine NAAs are different. When the affinity ratio of methionine to the nine NAAs is defined as Km(i)/Km, where Km is the half-saturation constant for methionine and Km(i) is the half-saturation constant for each NAA, the concentration Ci of each NAA divided by the Km(i)/Km is the concentration corresponding to methionine for the NAA carrier system. The sum of Ci/(Km(i)/Km) for the nine NAAs, a weighted sum of the NAAs (C’), is the methionine-equivalent concentration of the nine NAAs for the carrier system. The Km(i) in the rat brain was used as follows: methionine 0.040, phenylalanine 0.011, tryptophan 0.015, isoleucine 0.056, leucine 0.029, valine 0.21, threonine 0.22, tyrosine 0.040, and histidine 0.100 (μmole/ml) [29]. The weighted sum of the plasma concentrations of the NAAs determined after the volunteers had fasted for 4 h was calculated and examined in relation to the SUV max of each organ in the fourth scanning at 19.6 to 24.1 min.

### Statistical analysis

The relationship between the SUV max of each organ in the fourth scanning at 19.6 to 24.1 min and the weighted sum of the plasma concentrations of the NAAs determined after the volunteers had fasted for 4 h was analyzed in every part with linear regression and Spearman correlation tests. In all statistical analyses, significance was defined as a *P* value less than 0.05. Statistical analysis was performed with StatMate IV (ATMS Co., Ltd., Tokyo, Japan).

## Results

### Evaluation of the biodistribution

**Figure 1:**
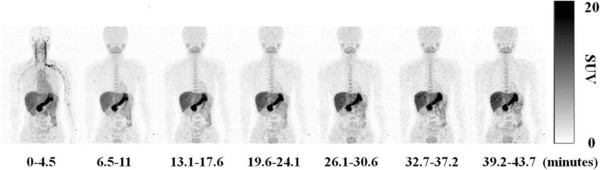
**Whole-body coronal PET images.** Representative whole-body decay-corrected coronal maximum-intensity projection images of a 24-year-old healthy male after injection of MET. High accumulation in the pancreas and liver were observed soon after the injection and persisted over time.

Figure 1 illustrated whole-body distribution of MET after venous injection. MET activity in the vessels was depicted only from 0 to 4.5 min and was rapidly cleared thereafter. MET accumulated preferentially in the pancreas and liver immediately after the injection, and the radioactivity also persisted at high levels subsequently in the pancreas and liver. Moderate accumulation in the glandular system, such as in the parotid glands and bone marrow, was also observed. The radioactivity of the urine in the urinary bladder gradually increased and reached a plateau at 26.1 to 30.6 min after the MET injection. MET accumulation in the brain, lung, and muscle was low throughout the imaging period. The blood activity was low even from 6.5 to 11 min, suggesting rapid blood clearance.

The average SUV max levels in 18 source organs were obtained from the seven repeated whole-body PET measurements in 10 of the 11 subjects and six repeated whole-body PET measurements in the remaining 1 subject. The radioactivity of the blood pool in the left ventricle decreased immediately during 6.5 to 11 min, suggesting rapid blood clearance. The SUV max in the pancreas reached a plateau from 6.5 to 11 min after the MET injection (SUV max = 25). The SUV max in the brain, lung, and myocardium reached a plateau from 19.6 to 24.1 min after the radiotracer injection (SUV max = 3, 1, and 4, respectively). The MET uptake in the spleen and kidney peaked initially early after the injection and decreased during this period. Urinary radioactivity in the urinary bladder reached a plateau from 26.1 to 30.6 min after the MET injection. The MET uptake in the liver and stomach wall rapidly increased during the first 0 to 4.5 min and gradually elevated thereafter during the scan period. The average SUV max levels in each organ are shown in Table 2.

**Table 2:** Average SUV max levels in each organ

	**0 to 4.5 min (*n *= 11)**	**6.5 to 11 min (*n *= 11)**	**13.1 to 17.6 min (*n *= 11)**	**19.6 to 24.1 min (*n *= 11)**	**26.1 to 30.6 min (*n *= 11)**	**32.7 to 37.2 min (*n *= 11)**	**39.2 to 43.7 min (*n *= 10)**
Brain	2.1 ± 1.3	2.5 ± 0.6	2.7 ± 0.7	2.8 ± 0.4	2.8 ± 0.7	3.2 ± 0.5	3.0 ± 0.9
Parotid	7.8 ± 4.2	6.1 ± 1.3	5.9 ± 1.3	5.7 ± 1.5	6.0 ± 1.5	5.3 ± 1.7	5.9 ± 2.2
Lung	2.8 ± 0.9	1.2 ± 0.2	1.1 ± 0.3	1.1 ± 0.3	1.2 ± 0.3	1.3 ± 0.4	1.4 ± 0.5
Myocardium	9.0 ± 3.0	4.2 ± 0.8	3.9 ± 0.9	4.0 ± 1.0	3.7 ± 0.9	3.7 ± 0.7	3.8 ± 1.2
Ventricular blood pool	9.0 ± 3.1	3.0 ± 0.2	2.8 ± 0.8	2.5 ± 0.5	2.6 ± 0.7	2.6 ± 0.6	2.6 ± 0.6
Stomach	15.4 ± 6.1	13.9 ± 4.8	16.1 ± 4.8	16.5 ± 5.5	16.6 ± 5.1	18.5 ± 5.3	17.9 ± 5.4
Spleen	11.2 ± 2.2	6.4 ± 2.2	6.5 ± 2.8	6.2 ± 2.7	5.9 ± 1.8	6.7 ± 2.5	6.3 ± 1.6
Liver	14.5 ± 3.8	12.9 ± 2.2	14.0 ± 2.3	15.1 ± 2.5	15.6 ± 2.0	16.6 ± 2.5	17.2 ± 2.3
Pancreas	30.1 ± 8.3	24.5 ± 5.1	25.9 ± 5.7	26.5 ± 6.7	24.8 ± 6.9	26.5 ± 7.9	24.4 ± 7.0
Kidney	14.4 ± 4.6	7.1 ± 1.9	6.7 ± 1.3	6.8 ± 1.5	6.6 ± 2.1	7.1 ± 2.1	6.7 ± 1.8
Intestine (right)	5.9 ± 2.4	4.9 ± 2.0	4.9 ± 1.8	5.3 ± 2.0	5.2 ± 2.0	5.3 ± 2.3	4.8 ± 2.3
Intestine (left)	6.1 ± 3.5	4.8 ± 2.9	5.1 ± 3.5	5.5 ± 3.4	4.8 ± 2.2	5.3 ± 2.6	5.6 ± 2.8
Bladder	3.7 ± 1.7	7.4 ± 5.4	10.3 ± 5.4	11.2 ± 5.9	12.8 ± 5.5	12.0 ± 5.9	11.5 ± 4.5
Muscle	2.2 ± 0.7	1.9 ± 0.6	2.1 ± 0.5	2.4 ± 0.7	2.5 ± 0.4	2.2 ± 0.4	2.7 ± 0.8
Bone marrow	6.5 ± 2.2	5.0 ± 1.2	5.4 ± 1.2	5.7 ± 1.3	6.3 ± 1.2	6.6 ± 1.9	5.8 ± 1.3
Prostate^a^	6.0 ± 1.9	4.7 ± 1.9	5.0 ± 1.7	4.9 ± 1.2	6.4 ± 2.6	5.3 ± 2.0	4.6 ± 1.0
Breast^b^	1.2 ± 0.0	1.0 ± 0.1	0.8 ± 0.1	1.0 ± 0.4	1.1 ± 0.2	1.3 ± 0.1	2.0 ± 0.9
Uterus^b^	5.9 ± 0.3	4.0 ± 0.3	3.4 ± 0.1	3.7 ± 0.2	3.9 ± 0.5	3.7 ± 0.1	6.6 ± 1.1

Values are means ± SD. ^a^Nine male volunteers were included in the evaluation. ^b^Two female volunteers were included in the evaluation.

### Measurement of NAA concentrations in the plasma

**Figure 2:**
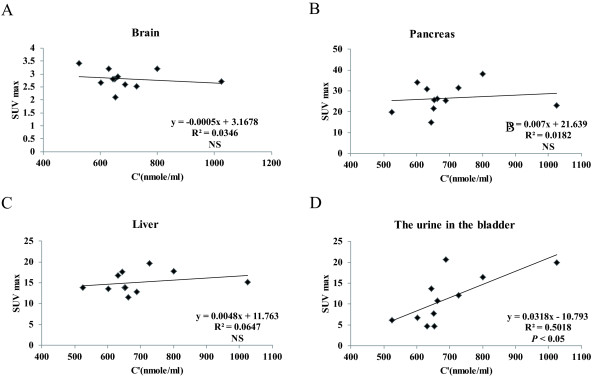
**Correlation between the C’ and the SUV max in representative organs of the fourth scanning.** No significant negative correlations were observed between the C’ and SUV max in the brain, pancreas, or liver in the fourth scanning (19.6 to 24.1 min) in the 11 volunteers (**A**, **B**, **C**). On the other hand, a significant correlation was observed between the C’ and SUV max in the urine contained in the urinary bladder in the fourth scanning (**D**).

The mean plasma concentrations of each of the nine NAAs in normal adults measured after the subjects had fasted for 4 h are shown at Table 3. Despite some unevenness found in these values, the plasma levels of all nine NAAs in the 11 subjects measured after 4-h fasting were within the normal range. The weighted sum of the NAA plasma concentrations measured in the 11 subjects after they had fasted for 4 h was stable at 525.2 to 1,025.2 nmol/ml (Table 4). There was a significant correlation between the weighted sum of the NAA plasma concentrations and the time at which the SUV max of the urine in the urinary bladder in the fourth scanning reached a plateau (*P* < 0.05). No such significant correlation was observed in the other organs. These relationships in representative organs are shown in Figure 2.

**Table 3:** Mean plasma concentration of each NAA

**NAA**	**Eleven subjects (nmol/ml)**	**Normal range (nmol/ml) (95% CI)**
Methionine	26.5 ± 7.4	18.9 to 40.5
Phenylalanine	54.7 ± 8.4	42.6 to 75.7
Tryptophan	44.2 ± 9.0	37.0 to 74.9
Isoleucine	68.6 ± 21.1	43.0 to 112.8
Leucine	118.9 ± 33.3	76.6 to 171.3
Valine	223.2 ± 40.2	147.8 to 307.0
Threonine	131.5 ± 33.6	66.5 to 188.9
Tyrosine	60.2 ± 7.1	40.4 to 90.3
Histidine	78.9 ± 7.9	59.0 to 92.0

Values are means ± SD. CI, confidence interval.

**Table 4:** Weighted sum of NAA plasma concentrations for each subject

**Subject number**	**Age (years)/sex**	**C’ (nmol/ml)**
1	24/M	687.9
2	26/M	800.2
3	24/M	651.8
4	24/M	1025.2
5	24/M	661.8
6	24/M	601.9
7	25/M	726.6
8	24/M	630.7
9	25/M	525.2
10	24/F	644.8
11	24/F	654.1

C’, weighted sum of the plasma concentrations of the nine NAAs.

## Discussion

The present study revealed dynamic changes in the radioactivity in the whole body for 43.7 min after MET infusion in normal volunteers. The radioactivity reached a plateau within 6.5 to 11 min in the pancreas and within 19.6 to 24.1 min in the brain, lung, and myocardium. The radioactivity, as represented by the SUVs, was not affected by the NAA concentrations in the plasma if the PET study was conducted in the subjects after a fasting period of 4 h.

In the 2-[^18^F]fluoro-2-deoxy-D-glucose (FDG) PET study, while the SUVs decline in normal tissues in the post-injection period, those in tumors generally increase, indicating that the SUVs of normal and tumor tissues are dependent on the time of scan initiation after infusion. Therefore, it is important to initiate the scanning at a consistent time-point after the FDG injection. The SNM procedure guidelines for tumor PET imaging recommended that emission images should be obtained at least 45 min after injection of the radiopharmaceutical [30]. The EANM procedure guidelines for tumor PET imaging recommended a 60-min interval between FDG administration and the start of image acquisition [31]. In contrast, MET accumulation in MET PET reaches a plateau in most normal organs. The MET accumulation in brain tumors and lung cancers also reached a plateau at 5 to 10 min after the radiotracer injection [32,33]. These findings imply that after the plateau phase is reached, the SUVs of normal tissues and tumors in MET PET might be independent of the scan initiation time. In most previous studies, the scanning was initiated 15 min or later after MET injection (Table 1). Therefore, in accordance with the above idea, their SUV values were consistent, despite the differing scan initiation times. It is noteworthy that the SUV values of brain tumors and lung cancers measured at 20 min after MET injection were significantly correlated with the uptake values estimated by repeated PET scan after MET infusion, metabolite-corrected arterial input function, and graphic analysis [34].

The radioactivity half-life of MET is short, being 20.4 min. If the PET scan can be initiated earlier than this time after injection of MET, high radioactivity can be expected, and much information can be acquired. Even with small doses of MET, a large number of patients can be evaluated by MET PET-CT within a short time. This also leads to reduction of the patient’s exposure to radioactivity. Our results show that MET PET scanning for evaluating the aforementioned targeted organs may be started 20 min or later after injection of MET. However, even if SUV levels are not stable in all organs, in several organs such as the pancreas, spleen, and kidney, the scan initiation from 15 min later does not seem to become the problem.

Another factor influencing the SUVs of MET is the plasma concentrations of NAAs. [^11^C]MET is transported through the NAA transporter from the plasma to the tissues. Therefore, plasma NAA concentrations could affect the uptake of [^11^C]MET in a competitive fashion. Ito et al. reported that brain uptake of the NAA tracer [^18^F]fluorophenylalanine was inversely correlated with the plasma NAA concentrations [29]. However, no previous studies have investigated the effects of plasma NAA concentrations on [^11^C]MET accumulation in the target tissues. In this study, we did not find a significant effect of the plasma methionine-equivalent concentrations on the MET uptake. This is considered to be due to the fact that in our subjects, the NAA concentrations in the plasma returned within normal range after a fasting period of 4 h (525.2 to 1,025.2 nmol/ml). On the other hand, Ito et al. did not set a fasting period prior to the study [29]. Four of the 14 patients in their study showed plasma NAA concentrations outside the normal ranges.

There are two limitations to this study. First, though blood clearance was one of the important factors for determining the best timing of scan initiation, we did not take blood samples and measure their radioactivities during the scan. We carefully set and measured ROI in the left ventricle blood pool instead and confirmed that blood clearance was rapid. Second, [^18^F]fluorophenylalanine mainly recognizes amino acid transport but is not metabolized in the cell, while MET is metabolized in the cells after transportation. The difference may affect the effect of NAA on tissue MET uptake.

## Conclusions

The present study demonstrated that the SUVs of MET in normal tissues can be measured by starting the PET imaging 20 min or later after MET infusion, during the plateau phase of accumulation, and by requesting a 4-h fasting period prior to the study. These conditions for the procedure are expected to provide the SUVs of MET in the target tissues independent of the scan initiation timing or the plasma NAA concentrations.

## Competing interests

The authors declare that they have no competing interests.

## Authors’ contributions

KI, ES, and JH participated in the design of the study and performed the statistical analysis. Data acquisition was done by KI, ES, YK, SN, and KF. Data analysis was done by KI, HK, and HW. The manuscript was prepared by KI. All authors read and approved the final manuscript.
